# Report of the Committee Appointed by the Right Hon. the Governor of Bengal for the Establishment of a Fever Hospital, and for Inquiring into Local Management and Taxation in Calcutta

**Published:** 1843-07

**Authors:** 


### Art. XIII.

*Report of the Committee appointed by the Right Hon. the Governor of Bengal for the establishment of a Fever Hospital, and for inquiring into Local Management and Taxation in Calcutta.—Calcutta,* 1839. Folio, pp. 245.

The recent inquiries into the sanatory condition of our own cities, of which due notice has been taken in this Journal, have naturally led us to look at what has been done elsewhere; and in the course of our investigation, we have been much gratified in observing the great attention which has been recently bestowed on the sanatory improvement of the metropolis of British India. The document now before us contains the suggestions of a committee of eminent and highly competent individuals, relative to the measures which they deem conducive to the removal of the causes of endemic disease, and the efficient relief of the poorer classes suffering from its effects.

The length at which we have entered on the kindred subject of Mr. Chadwick's reports, forbids our bestowing more than a cursory notice on that of the Calcutta committee. To the energy and benevolence of Mr. Martin, the late surgeon of the Calcutta Native Hospital, is due the appointment of the committee. The attention and zealous industry evinced by that body in their investigations is beyond all praise, and although few if any of their suggestions have been acted upon by the government, nevertheless their inquiries have at least done this good, that individual benevolence has been excited to acts of munificent charity, and some serious local nuisances have been considerably abated.

Scattered throughout the report, we find numerous proofs of the correct and enlightened views with which Mr. Martin directed the labours of the committee. We are induced to select the following passages in justification of the favorable opinion we have formed of the exertions of this active officer, in forcing on the notice of the government and the public, the measures he deemed essential to the preservation of the general health. On the result of local improvement as affecting the intensity of endemic remittent fever in Bengal, Mr. Martin has the following observations:

"The causes of the present superiority in public health must be of the highest interest and importance, especially to communities living within the tropics; and with all the just confidence in modern medicine, guided by the lights of an improved physiology and those of pathology, I cannot yet agree with those who would ascribe the *whole* of the difference here spoken of to superior modes of medical management, great as these confessedly are. It is not through the advantages of modern improvement in the treatment of mere disease, as contrasted with the more ancient modes, that the public health has been so much amended, as through the great measures of the *prevention of disease* consequent on the progress of the public mind, and of governments, *general knowledge*, leading directly to improved habits of life in communities, improved localities, institutions of police, &c.; it is to the preservative power of knowledge, to the reciprocal actions of the social state, and of political events upon each other, and upon medical science, that the advancement of public health is most indebted and that it will continue to be so, although the circumstances are not sufficiently weighed by some of us, when in our hurry to praise ourselves, we forget what is due to our predecessors, and that these last had frequently to treat a form of disease which we have never seen, and with whose fatal severity we are, consequently, unacquainted."

Again, when speaking of epidemics, and of the influence of local improvements on their spread and intensity, Mr. Martin observes:

"A history of our local epidemics would be of great value, as enabling us to trace their connexion with changes of climate and condition of the surrounding localities, or with the social condition of the people, both European and native; had such a history existed, it would have helped to an earlier establishment of general principles, and a rational plan of treating our fevers especially; for, though all the epidemics within my personal recollection (and there is scarcely a year we have not one in some form) differ greatly from the ordinary endemics of the country, still, there will be found in most of them, so much of the savour of the soil, if I may be allowed the expression, as to render a knowledge of their history and treatment an object of no mean importance. It is these sweeping epidemics, aggravated by endemial and social influences, that swell out our bills of mortality, and that did so in former times especially, to such a frightful extent.

"Under a system of local improvement calculated to diminish the endemic sources of disease, I am satisfied that the value of European life in this city (Calcutta) would be greatly enhanced.

"From what is here said, however, it must not be supposed that we are without help in the case of epidemics, or that our means of prevention apply only to such diseases as are peculiar to the climate; far otherwise is the fact. Even epidemics are greatly modified by states of locality; and, as stated in another place, *they are found to fasten with peculiar severity, and remain longest, in such localities as are neglected;* in truth endemics are very often the parent stock upon which epidemics are engrafted. Why is it we have not now, as formerly, those terrible epidemic fevers which swept off '800 Europeans, and 50,000 blacks?' The cause is obvious enough to the most ordinary understanding; it is the same that has banished the former malignant intermittents of our city, to which we owe our present comparative exemption; in short, when filth, want, and misery are removed, epidemics will have lost their chief power. It is these that everywhere give such destructive influence to climate, and that gave rise to the observation of Cabanis, 'que l'effet du climat n'est pas le même pour le riche que pour le pauvre.' The impure state of London during the seventy-three years from 1592 to 1666 was such, that the average deaths in each year, from the plagues, amounted to about a fourth part of the whole population; or what would, for the present population, amount to the fearful sum of 375,000 persons per annum! Owing to the modern improvements of London again, only one person died out of 250 inhabitants, or little more than 6000 in the worst year of cholera, the severest plague which has visited London since 1666."

It is unnecessary to prolong this notice, as the details embodied in the report are so strictly local in their nature, that little general benefit could flow from their consideration. We have pleasure in reiterating our sense of the great value of Mr. Martin's labours. We are bound, too, to express our hope that he may find many to follow his example in toiling to improve the public health in the great and populous cities of British India, of which so many are still abandoned to the destructive influence of the jungle and morass, and are at the same time destitute of all efficient means of relief for the sufferers from such unpardonable neglect. Mr. Martin himself has, however, abandoned his old field of exertion, and transferred his services from the metropolis of India to that of England, where, we doubt not, he will find his talents appreciated as they deserve, both by the profession and the government.

